# Persistent Methicillin-Susceptible Bacteremia Rapidly Cleared with Cefazolin and Ertapenem Combination Therapy in a Patient with COVID-19

**DOI:** 10.1155/2022/6828538

**Published:** 2022-04-20

**Authors:** Dan Ilges, Gayathri Krishnan, Elvin Geng

**Affiliations:** ^1^Mayo Clinic Hospital, Phoenix, AZ, USA; ^2^Washington University School of Medicine, St. Louis, MI, USA

## Abstract

Methicillin-susceptible *Staphylococcus aureus* (MSSA) bloodstream infections (BSIs) are associated with significant morbidity and mortality. MSSA BSIs can rapidly disseminate, resulting in deep-seated infections, prolonged durations of bacteremia, and further metastases. Recently, cefazolin and ertapenem combination therapy has emerged as a potential therapeutic strategy to sterilize the blood in patients with persistent MSSA bacteremia. Here, we present a patient with COVID-19 pneumonia and concomitant MSSA BSI achieving blood culture sterilization within 24 hours of cefazolin and ertapenem combination therapy initiation following 11 days of positive blood cultures.

## 1. Introduction

*Staphylococcus aureus* is a leading cause of bloodstream infections (BSIs), with an annual incidence in the United States of approximately 30 cases per 100,000 person-years [1]. *S. aureus* BSI is associated with high morbidity and mortality, with 30-day all-cause mortality rates ranging from 20 to 30% [2, 3]. In recent years, MSSA has once again emerged as the predominant cause of staphylococcal bacteremia [4]. Thus, optimizing treatment strategies for MSSA BSI is paramount.

The treatment of choice for MSSA BSI is source control along with an antistaphylococcal penicillin, such as oxacillin or nafcillin or cefazolin [5]. Persistent bacteremia, defined anywhere from 2 to 7 days of positive blood cultures despite appropriate antibiotic therapy, is associated with worse outcomes and higher mortality compared to bacteremias that clear rapidly [6, 7]. Unfortunately, modifications to antibacterial therapy, including the addition of rifampin [8] and daptomycin [9], have not proven beneficial in patients with MSSA BSI. The addition of gentamicin has been used historically but is no longer recommended due to an unacceptable risk of nephrotoxicity when combined with antistaphylococcal penicillins [5].

Recently, combination therapy with cefazolin and ertapenem has emerged as a possible rescue therapy in patients with persistent MSSA BSI. The combination demonstrated synergy both *in vitro* and *in vivo* in a murine skin infection model [10]. In a small case series, combination therapy was found to sterilize blood cultures within 24 hours of initiation in 8 of 9 patients with daily blood culture samples [11]. In another case report of a neonate with eight days of positive blood cultures, escalating to cefazolin and ertapenem preceded negative blood cultures 30 hours later [12]. More data, however, about clinical outcomes are needed. Here, we describe a case in which blood culture sterilization was observed within 24 hours of initiation of cefazolin and ertapenem combination therapy after 11 days of persistent bacteremia on oxacillin in a patient with MSSA BSI and COVID-19.

## 2. Case Description

A 45-year-old male with a past medical history significant for obstructive sleep apnea, congenital club foot status post corrective surgery years prior, and recently diagnosed COVID-19 (10 days prior to admission) presented to the emergency department with increasing weakness, fatigue, and loss of appetite. The patient got tested for COVID-19 after his partner developed respiratory symptoms. Respiratory symptoms at home included cough, shortness of breath, and fevers. The patient also reported using his continuous positive airway pressure machine during the day for increased dyspnea. Additionally, he reported progressive right ankle pain and redness over the last week. On physical exam, the right ankle was warm and erythematous without gross effusion and was tender to palpation and passive range of motion. Initial vital signs were remarkable for a respiratory rate of 28 breaths/minute, heart rate of 116 beats/minute, blood pressure of 184/96 mm·Hg, and a body mass index of 40 kg/m^2^. Complete metabolic panel revealed an elevated glucose of 462 mg/dL, blood urea nitrogen of 80 mg/dL, and serum creatinine of 5.40 mg/dL (estimated creatinine clearance 23 ml/min). Additional labs were remarkable for HgA1c of 7.2%, NT-proBNP of 867 pg/mL, CRP of 329.3 mg/dL, ESR of 103 mm/hr, lactate of 2.7 mmol/L, and a D-dimer of 4623 mcg/mL. Blood and urine cultures were sent. Chest X-ray showed bilateral patchy airspace and interstitial opacities, predominantly involving the periphery of the right lung. Right ankle/foot X-ray showed findings consistent with prior congenital foot surgery without osseous erosions or fractures. He received 1 L lactated ringers, 5 units of lispro, and a dose of intravenous vancomycin 1750 mg before transfer to the medical intensive care unit (MICU) for BiPAP.

**Figure 1:**
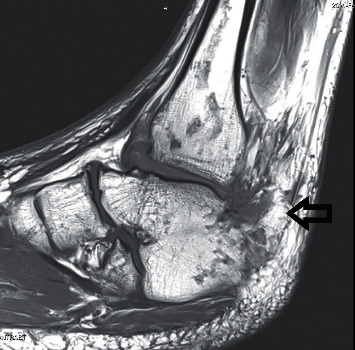
MRI of right ankle showing large complex tibiotalar joint effusion with synovitis and associated fluid collection along the flexor hallucis longus muscle belly (day 3 of hospitalization).

Complete details of the hospital course are provided in Table 1. The patient was initially started on intravenous dexamethasone and remdesivir in the MICU. Antimicrobial therapy was changed to oxacillin on day 2 following the detection of MSSA in initial blood cultures via Verigene Blood Culture Nucleic Acid Test. Infectious disease was consulted the following day in accordance with institutional practice for all patients with staphylococcal BSIs. An MRI performed on the right ankle revealed diffuse skin thickening and edema of foot and ankle without evidence of OM, large complex tibiotalar joint effusion with synovitis, and abscess along the flexor hallucis longus muscle (Figure 1), which was urgently debrided in the operating room. The following day, the patient was weaned from BiPAP back to CPAP at night only.

**Figure 2:**
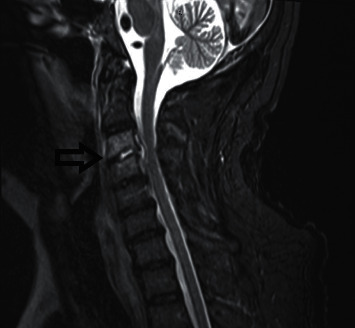
MRI of spine with and without contrast showing C_3_-C_4_ discitis-osteomyelitis (black arrow), with associated epidural abscess (day 9 of hospitalization).

Despite initial source control and appropriate antimicrobial therapy, blood cultures remained positive. Echocardiographic imaging on days 2 and 7 did not reveal any valvular vegetations; yet, the patient remained persistently bacteremic despite therapy with intravenous oxacillin. On day 9 of admission, the patient was escalated to intravenous cefazolin and ertapenem combination therapy and dexamethasone was stopped with the completion of 10 doses. On the same day, an MRI spine revealed discitis/osteomyelitis of the cervical spine with associated epidural abscess (Figure 2). The patient was taken for urgent debridement and C_3_ to C_4_ cervical fusion. Intraoperative spinal cultures grew MSSA. Repeat blood cultures within 24 hours of administration of cefazolin and ertapenem combination therapy finalized without growth. Follow-up cultures remained sterile through hospital discharge on day 19. The patient was discharged to a long-term acute care facility to complete 6 weeks of intravenous cefazolin and ertapenem from the first negative blood culture.

## 3. Discussion

MSSA bacteremia is a common BSI associated with significant morbidity and mortality [2, 3]. Effective management includes source control and treatment with antistaphylococcal penicillin or cefazolin. Limited therapeutic options exist for recalcitrant MSSA BSIs not responding to standard therapy and source control.

Here, we report on a patient with newly diagnosed diabetes mellitus, COVID-19 pneumonia, and concomitant MSSA BSI not responding to the standard of care therapy with oxacillin and initial source control procedures. Following more than 10 days of persistent bacteremia, blood cultures were sterilized within 24 hours of administration of cefazolin and ertapenem combination therapy.

Cefazolin and ertapenem bind to different penicillin-binding proteins (PBPs), with ertapenem selectively targeting PBP1 and cefazolin predominantly binding to PBP2. [13–15] Such complementary binding affinities provide the theoretical pharmacodynamic foundation for utilizing these two agents in combination. A recent case series highlights the potential utility of cefazolin and ertapenem in combination as salvage therapy for persistent MSSA BSIs [11]. In this series, 8 of 11 patients achieved negative blood cultures within 24 hours of initiation of salvage therapy. Most patients in this series had deep-seated infections, including 6 with endocarditis and 2 with spinal osteomyelitis and epidural abscesses. Additionally, the authors noted synergy when testing the combination *in vitro* and in a rat model of endocarditis [11]. While the duration of *S*. *aureus* bacteremia has not been associated with 90-day mortality, it has been associated with other significant complications. These include new metastatic foci of infection (often requiring surgical interventions), relapse of bacteremia, longer hospital stay, and complications due to longer hospital stay [7, 16]. Together, these serve as a rationale for salvage therapy with combination antimicrobials in recalcitrant bacteremia.

Regarding the management of the patient's COVID-19 pneumonia, our patient initially received intravenous dexamethasone and remdesivir; however, the latter was held following the administration of a loading dose given the patient's acute renal failure. Dexamethasone was continued for a complete 10-day course in accordance with clinical treatment guidelines [17]; however, new initiation of corticosteroids in patients with staphylococcal BSIs without concomitant shock has been associated with increased all-cause mortality at 28 and 90 days [18]. These data highlight the need to critically weigh the risks and benefits of continued dexamethasone therapy in the setting of secondary infections in patients with COVID-19.

One final consideration is the timing of source control relative to the imitation of cefazolin and ertapenem combination therapy. As a case report, we cannot be certain of the causal link between the antibiotic change and the cessation of bacteremia, but the temporal proximity does raise this as a possibility. In addition, while an undrained epidural abscess could explain persistent fevers and symptoms, undrained collections are rarely the cause of continuous bacteremia (hence, our clinical use of continuous bacteremia as the sine qua non of endovascular infection). In total, this case suggests to us the possibility of an effect, and one worth reporting as a case study, in order to pique the interest of other clinicians or to contribute eventually to a case series, before undertaking a more rigorous study design.

## 4. Conclusion

Cefazolin and ertapenem combination therapy resulted in successful sterilization of blood cultures within 24 hours of administration in a patient previously bacteremic for more than 10 days despite optimal pharmacotherapy. The patient's MSSA BSI may have been aggravated by continued administration of dexamethasone for COVID-19. Clinicians should consider weighing the risks and benefits of dexamethasone administration in the setting of concomitant infections.

## Figures and Tables

**Table 1:** Summary of hospital course.

Day	*T*_max_	WBC	SCr	Cultures	Antimicrobials	Imaging	Comments
ED	40.5	8.0	5.10	Bcx: 2/2 MSSA; TTP 8.3 hours	Vancomycin 1750 mg IV *×* 1	X-ray R foot/ankle, X-ray chest per HPI	—
1	37.9	8.1	4.44	Bcx: 2/2 MSSA	Cefepime 2 g IV q24 h, linezolid 600 mg IV q12 h, remdesivir 200 mg IV *×* 1	CT chest PE protocol: no PE, possible septic emboli and L IJ thrombophlebitis	Ortho attempted aspiration of R ankle—dry tap. Oxacillin 2 g IV q4 h started following detection of MSSA in ED blood cultures.
2	37.3	8.7	4.19	Bcx: 2/2 MSSA	Oxacillin 2 g IV q4 h	Panorex: periapical abscesses at ^#^30. Duplex LEs: No DVTs. TTE: No vegetations	Remdesivir stopped by MICU due to AKI. ID consulted.
3	37.3	7.6	2.53	Ankle tissue culture: 5/5 MSSA; tracheal aspirate: MSSA		MRI R ankle (Figure 1): diffuse skin thickening and edema of foot and ankle w/o evidence of OM; large complex tibiotalar joint effusion w/synovitis and abscess along the FHL muscle	OR with ortho s/*p* R ankle I&D and arthrotomy; R leg FHL abscess I&D. Dental consulted.
4	38.5	5.3	2.12	Bcx: 2/2 MSSA		X-ray chest: mild interval improvement in b/l interstitial airspace opacities c/w COVID-19 pneumonia, septic pulmonary emboli	Weaned off BiPAP; CPAP at night only. Tooth #30 extracted.
5	38.8	5.7	1.82	Bcx: 2/2 MSSA			
6	38.9	6.2	1.57	Bcx: 2/2 MSSA; COVID-19 RNA nasopharyngeal swab: positive	Oxacillin 2 g IV q4 h, ceftriaxone 2 g IV × 1	CT CAP w/contrast: cavitating pulmonary septic emboli, fluid collection around L SC joint c/*f* early septic arthritis, phlegmon within R iliacus muscle. X-ray L knee/ankle: L knee medial osteoarthritis, L foot cellulitis, and acute on chronic achilles tendinopathy	Ceftriaxone added by ICU team for Gram-negative coverage given fever and tachycardia; X-rays looking for prior hardware; no surgical indication for SC joint per thoracic surgery.
7	38.7	4.2	1.47	Bcx: 2/2 MSSA	Oxacillin 2 g IV q4 h, ceftriaxone 1 g IV q24 h	Tee: no vegetations noted	MSK IR consulted for iliacus muscle phlegmon.
8	37.9	7.1	1.51	Bcx: 1/1 MSSA		X-ray L shoulder/R knee: no evidence of OM or septic arthritis. MRI L knee/ankle/foot: severe L knee chondrosis, small L knee joint effusions, L ankle and foot cellulitis, small nonspecific collection extending dorsally from between the base of the L 3^rd^ and 4^th^ metatarsal, likely represents extension of adventitial bursitis. US lower back: loculated fluid collection deep and posterior in pelvis. MRI brain/spine (Figure 2): C_3_-C_4_ discitis/OM w/epidural abscess, no intracranial abnormality. CT spine: C_3_-C_4_ discitis/OM w/epidural abscess	Transferred to floor.
9	37.7	—	—	Bcx: 1/1 MSSA; TTP = 13.6 hours			Ceftriaxone discontinued. ID recommend to change oxacillin to cefazolin plus ertapenem. Last dose dexamethasone given. Ortho spine consulted.
10	37.2	7.1	1.39	Bcx: 2/2 MSSA; TTP = 22.5 hrs; L foot aspiration: MSSA; cervical spine wound: MSSA	Cefazolin 2 g IV q8 h, ertapenem 1 g IV q24 h		
11	36.9	6.0	1.23	Bcx: 2/2 no growth			L 3rd/4th metatarsal aspiration; OR for cervical fusion/epidural abscess drainage with ortho spine.
12	37.2	4.1	1.10	Bcx: 2/2 no growth		—	—

Bcx, blood culture(s); TTP, time-to-positivity; FHL, flexor hallucis longus; SC, sternoclavicular; OM, osteomyelitis.

## References

[1] Lam J. C., Gregson D. B., Robinson S., Somayaji R., Conly J. M., Parkins M. D. (2019). Epidemiology and outcome determinants of *Staphylococcus aureus* bacteremia revisited: a population-based study. *Infection*.

[2] Shurland S., Zhan M., Bradham D. D., Roghmann M.-C. (2007). Comparison of mortality risk associated with bacteremia due to methicillin-resistant and methicillin-susceptible *Staphylococcus aureus*. *Infection Control & Hospital Epidemiology*.

[3] Inagaki K., Lucar J., Blackshear C., Hobbs C. V. (2019). Methicillin-susceptible and methicillin-resistant *Staphylococcus aureus* bacteremia: nationwide estimates of 30-day readmission, in-hospital mortality, length of stay, and cost in the United States. *Clinical Infectious Diseases: An Official Publication of the Infectious Diseases Society of America*.

[4] Diekema D. J., Pfaller M. A., Shortridge D., Zervos M., Jones R. N. (2019). Twenty-year trends in antimicrobial susceptibilities among *Staphylococcus aureus* from the SENTRY antimicrobial surveillance program. *Open Forum Infectious Diseases*.

[5] Baddour L. M., Wilson W. R., Bayer A. S. (2015). Infective endocarditis in adults: diagnosis, antimicrobial therapy, and management of complications. *Circulation*.

[6] van Hal S. J., Jensen S. O., Vaska V. L., Espedido B. A., Paterson D. L., Gosbell I. B. (2012). Predictors of mortality in *Staphylococcus aureus* bacteremia. *Clinical Microbiology Reviews*.

[7] Kuehl R., Morata L., Boeing C. (2020). Defining persistent *Staphylococcus aureus* bacteraemia: secondary analysis of a prospective cohort study. *The Lancet Infectious Diseases*.

[8] Thwaites G. E., Scarborough M., Szubert A. (2018). Adjunctive rifampicin for *Staphylococcus aureus* bacteraemia (ARREST): a multicentre, randomised, double-blind, placebo-controlled trial. *Lancet (London, England)*.

[9] Cheng M. P., Lawandi A., Butler-Laporte G., De l’Etoile-Morel S., Paquette K., Lee T. C. (2020). Adjunctive daptomycin in the treatment of methicillin-susceptible *Staphylococcus aureus* bacteremia: a randomized controlled trial. *Clinical Infectious Diseases*.

[10] Sakoulas G., Olson J., Yim J. (2016). Cefazolin and ertapenem, a synergistic combination used to clear persistent *Staphylococcus aureus* bacteremia. *Antimicrobial Agents and Chemotherapy*.

[11] Ulloa E. R., Singh K. V., Geriak M. (2020). Cefazolin and ertapenem salvage therapy rapidly clears persistent methicillin-susceptible *Staphylococcus aureus* bacteremia. *Clinical Infectious Diseases*.

[12] Akers S. M., Kinney K., Butcher M. I., Moïse A. (2020). Clearance of persistent *Staphylococcus aureus* bacteremia in a preterm neonate with the use of combination cefazolin and ertapenem. *Journal of Pediatric Pharmacology and Therapeutics*.

[13] Chambers H. F., Sachdeva M. (1990). Binding of -lactam antibiotics to penicillin-binding proteins in methicillin-resistant *Staphylococcus aureus*. *Journal of Infectious Diseases*.

[14] Dumitrescu O., Choudhury P., Boisset S. (2011). *β*-Lactams interfering with PBP1 induce panton-valentine leukocidin expression by triggering sarA and rot global regulators of *Staphylococcus aureus*. *Antimicrobial Agents and Chemotherapy*.

[15] Bamberger D. M., Herndon B. L., Fitch J., Florkowski A., Parkhurst V. (2002). Effects of neutrophils on cefazolin activity and penicillin-binding proteins in *Staphylococcus aureus* abscesses. *Antimicrobial Agents and Chemotherapy*.

[16] Chong Y. P., Park S.-J., Kim H. S. (2013). Persistent *Staphylococcus aureus* bacteremia. *Medicine*.

[17] Bhimraj A., Morgan R. L., Shumaker A. H. (2020). Infectious diseases society of America guidelines on the treatment and management of patients with COVID-19. *Clinical Infectious Diseases*.

[18] Forsblom E., Nurmi A.-M., Ruotsalainen E., Järvinen A. (2016). Should all adjunctive corticosteroid therapy be avoided in the management of hemodynamically stabile *Staphylococcus aureus* bacteremia?. *European Journal of Clinical Microbiology & Infectious Diseases*.

